# Development of analytical methods to study the effect of malting on levels of free and modified forms of *Alternaria* mycotoxins in barley

**DOI:** 10.1007/s12550-022-00455-1

**Published:** 2022-04-08

**Authors:** Sophie Scheibenzuber, Fabian Dick, Marina Bretträger, Martina Gastl, Stefan Asam, Michael Rychlik

**Affiliations:** 1 Chair of Analytical Food Chemistry, Department of Life Science Engineering, School of Life Sciences, Technical University of Munich, Maximus-von-Imhof Forum 2, 85354 Freising, Germany; 2 Chair of Brewing and Beverage Technology, Department of Life Science Engineering, School of Life Sciences, Technical University of Munich, Freising, Germany

**Keywords:** Modified *Alternaria* toxins, Mycotoxins, Barley, Malt, LC–MS/MS analysis

## Abstract

A liquid chromatography tandem mass spectrometry (LC–MS/MS) multi-mycotoxin method was developed for the analysis of the *Alternaria* toxins alternariol (AOH), alternariol monomethyl ether (AME), tentoxin (TEN), altertoxin I (ATX I), altertoxin II (ATX II), alterperylenol (ALTP), and altenuene (ALT), as well as the modified toxins AOH-3-glucoside (AOH-3-G), AOH-9-glucoside (AOH-9-G), AME-3-glucoside (AME-3-G), AOH-3-sulfate (AOH-3-S), and AME-3-sulfate (AME-3-S) in barley and malt. The toxin tenuazonic acid (TeA) was analyzed separately as it could not be included into the multi-mycotoxin method. Quantitation was conducted by using a combination of stable isotope dilution analysis (SIDA) for AOH, AME, and TeA, and matrix-matched calibration for all other toxins. Limits of detection were between 0.05 µg/kg (AME) and 2.45 µg/kg (ALT), whereas limits of quantitation ranged from 0.16 µg/kg (AME) to 8.75 µg/kg (ALT). Recoveries between 96 and 107% were obtained for the analytes when SIDA was applied, while recoveries between 84 and 112% were found for analytes quantified by matrix-matched calibration. The method was applied for the analysis of 50 barley samples and their respective malts from the harvest years 2016–2020 for their mycotoxin content, showing the overall potential of toxin formation during the malting process. The toxins ALTP and ATX I were mainly found in the malt samples, but not in barley.

**Supplementary information:**

The online version contains supplementary material available at 10.1007/s12550-022-00455-1.

## Introduction

Black molds of the fungal species *Alternaria* spp. are ubiquitously present in the environment and are suspected to pose a risk to human health due to their ability to form various mycotoxins. Until now, over 100 different species of *Alternaria* have been identified with *A. alternata*, *A. citri*, *A. solani*, and *A. brassicae* being the most dominant ones (Woudenberg et al. 2013).

**Fig. 1:**
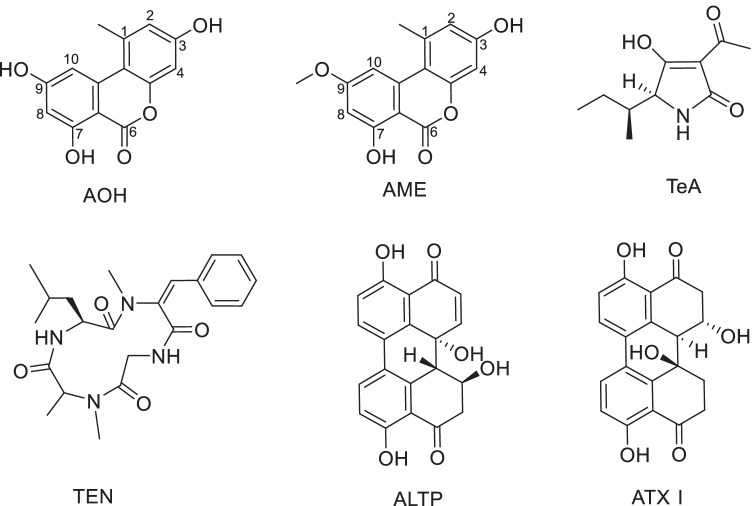
Chemical structures of the six most frequent *Alternaria* mycotoxins AOH, AME, TeA, TEN, ALTP, and ATX I

The most known *Alternaria* toxins are alternariol (AOH), alternariol monomethyl ether (AME), tenuazonic acid (TeA), tentoxin (TEN), altertoxin I (ATX I), altertoxin II (ATX II), alterperylenol (ALTP), altenuene (ALT), and stemphyltoxin III (STTX III) (Fig. 1). Also, the modified metabolites AOH-3-glucoside (AOH-3-G), AOH-9-glucoside (AOH-9-G), AME-3-glucoside (AME-3-G), AOH-3-sulfate (AOH-3-S), and AME-3-sulfate (AME-3-S) gained increased attention recently, as they might release their parent toxin during digestion and, therefore, contribute to the exposure level of AOH and AME (EFSA 2011).

Generally, the extent of mycotoxin production by fungi depends on external factors like temperature and water content (Lacey 1992), but also e.g. on the pH of the substrate, the infected kind of plant (Bottalico and Logrieco 1998; Lacey 1992), and the respective fungal species (Bottalico and Logrieco 1998; Grabarkiewicz-Szczesna and Chełkowski 1992). Concerning *Alternaria*, the species *A. alternata* was found to be the main mycotoxin producer whereas *A. solani*, *A. brassicae*, and *A. dauci* are mainly known to only produce AOH and AME (Bottalico and Logrieco 1998; Gotthardt et al. 2019a; Grabarkiewicz-Szczesna and Chełkowski 1992). Also, several studies have already demonstrated the ability of *Alternaria* spp. to grow in a temperature range between 1 and 35 °C and to produce mycotoxins between 10 and 35 °C, reaching maximal concentrations at 25–28 °C (Bottalico and Logrieco 1998; Lee et al. 2015; Magan and Lacey 1985). However, the exact temperature range can vary between different *Alternaria* species (Lacey 1992). In addition, higher humidity promotes both fungal growth and mycotoxin formation, with an *a*_w_ value of 0.90 and 0.98 as ideal condition for the formation of TeA and AOH, respectively (Magan and Lacey 1985).

The main food commodities that provide good growing conditions for *Alternaria* spp. are fruits, vegetables, and grains (Bottalico and Logrieco 1998; Grabarkiewicz-Szczesna and Chełkowski 1992; Logrieco et al. 2009; Scott 2001; Strandberg 1992). As barley is mainly used in its malted form in the brewing and bakery industry, *Alternaria* toxins should also be targeted in malt due to the elevated water content and temperatures during the malting process, which can promote fungal growth and mycotoxin production (Noots et al. 1999). The possibility of mycotoxin formation during this processing step was already shown for some *Fusarium* toxins (Habler et al. 2016), and therefore should be analyzed for other fungi as well.

To our knowledge, there is only limited data about *Alternaria* mycotoxins in malt published, yet. Therefore, we developed and validated a multi-mycotoxin liquid chromatography tandem mass spectrometry (LC–MS/MS) method for the analysis of 13 *Alternaria* mycotoxins in barley and malt. We then analyzed 50 barley samples and the respective standardized produced malts prepared from them to get a first insight into mycotoxin occurrence in barley and their possible formation during the malting process. In this study, the modified toxins AOH-3-G, AOH-9-G, AME-3-G, AOH-3-S, and AME-3-S were analyzed as well, which is quite important as data about those modified forms are still scarce and are only available for beer, sunflower oil, tomato products, cereals, fruits, and vegetables so far (Puntscher et al. 2018; Scheibenzuber et al. 2021; Walravens et al. 2014, 2016).

## Materials and methods

### Chemicals and reference standards

Water was purchased from Th. Geyer (Renningen, Germany), methanol (MeOH) from Honeywell Riedel-de Haën (Seelze, Germany), and acetonitrile (ACN) and the ammonia solution (25%) were purchased from VWR (Ismaning, Germany), all at least in analytical grade. Reference standards for TEN, ALT, and TeA were bought from Merck (Darmstadt, Germany), while AOH, AME, ATX I, ATX II, STTX III, ALTP, AOH-3-G, and AOH-9-G, AME-3-G as well as AOH-3-S and AME-3-S were either isolated out of fungal extracts or synthesized at our chair as described previously (Liu and Rychlik 2015; Scheibenzuber et al. 2020, 2021). The stable isotope–labelled standards [^2^H_4_]-AOH, [^2^H_4_]-AME, and [^13^C_6_, ^15^ N]-TeA were synthesized in our laboratory as reported in literature (Asam et al. 2009, 2011a).

### Preparation of stock solutions

All reference compounds were quantified by quantitative nuclear magnetic resonance (qNMR) measurements after their purchase, synthesis, or isolation as described by Frank et al. (2014). Afterwards, different stock solutions were prepared in either acetonitrile (TEN, ATX I, ATX II, ALT, ALTP, AOH-3-G, AOH-9-G, AME-3-G, STTX III) or methanol (AOH, AME, TeA, AOH-3-S, AME-3-S) in a concentration range from 1 to 100 µg/mL. To regularly verify the respective concentrations of the stock solutions, diluted standards were transferred into precision cells made out of quartz glass (Hellma GmbH & Co. KG, Müllheim, Germany) and measured with a UV spectrophotometer (Genesys, 10S, UV–Vis spectrophotometer, Thermo Fisher Scientific, Madison, WI, USA). Concentrations were then calculated by the respective molar extinction coefficients, which were either obtained from literature or determined in previous studies (Cole et al. 2003; Fleck 2014; Scheibenzuber et al. 2020; Scheibenzuber et al. 2021). Due to the instability of the analyte STTX III even when stored at − 80 °C, this toxin was only included qualitatively into the LC–MS/MS method.

### Samples

Fifty industrial malting barley samples from the harvest years 2016 to 2020 were analyzed before and after malting. Barley and malt samples from 2016 were finely ground and stored at − 18 °C prior to analysis in 2018, and all other samples were analyzed within a few weeks after sample receipt.

### Sampling of barley

Barley sampling at the various sample locations was conducted as specified by the Mitteleuropäische Brautechnische Analysenkommission (R-110.00.001 [2016–03], Jacob and Mitteleuropäische Brautechnische Analysenkommission 2011): using grain samplers, individual barley samples were taken from at least 10% of the grain bags and were combined to create a composite barley sample (CBS) of at least 5 kg, which was then sent to TUM. After arrival, a sample divider (Pfeuffer GmbH, Kitzingen, Germany) was used for all CBS to create representative laboratory barley samples of 0.5–1 kg (LBS). From each LBS, 50 g was taken as a subsample for mycotoxin analysis (mycotoxin analysis barley sample, MABS), respectively.

### Malting process

Malting was conducted every year shortly after sample receipt. One kilogram of each LBS was malted in a micro-malting procedure at the chair of brewing and beverage technology (TUM) according to MEBAK R-110.00.008 [2016–03], and standard malt parameters were analyzed based on the isothermal 65 C laboratory mashing regime R-207.00.002 [2016–03] (Jacob and Mitteleuropäische Brautechnische Analysenkommission 2011). In short, steeping was carried out at 14 °C for 48 h according to the following scheme: 5-h steeping, 19-h air rest, 4-h steeping, and 20-h air rest; the degree of steeping was 45%. After steeping, germination took place for 96 h at 14 °C, resulting in a green malt with a water content of 45%. Afterwards, kilning was conducted as follows: 16 h at 50 °C, 1 h at 60 °C, 1 h at 70 °C, and 5 h at 80 °C. The kilned malt was then cleaned to remove the rootlets from the grains. From each obtained malt, 50 g was taken as subsample for mycotoxin analysis (mycotoxin analysis malt sample, MAMS), respectively.

### Sample grinding

The whole amount (50 g) of each MABS and MAMS was ground in a laboratory mill (Grindomix GM200, Retsch GmbH, Germany) to a fine powder and then stored at − 18 °C until further use.

### Sample preparation for mycotoxin analysis

One gram of each sample was weighted in duplicate into a 50 mL centrifuge tube and spiked with 100 µL of a 0.1 µg/mL [^2^H_4_]-AOH solution and 100 µL of a 0.1 µg/mL [^13^C_6_, ^15^ N]-TeA solution, as well as with 100 µL of a 0.01 µg/mL [^2^H_4_]-AME solution. Then, 15 mL ACN/H_2_O (84/16, v/v) was added to the centrifuge tubes and samples were extracted on a horizontal shaker at 225 rpm for 1 h. After centrifugation for 5 min at 3220 g, the supernatant was transferred into a 50 mL pear-shaped flask. To the remaining residue, 15 mL of ACN/H_2_O (84/16, v/v) and 200 µL of formic acid were added, and samples were extracted on a horizontal shaker at 225 rpm for 30 min. Again, samples were centrifuged at 3220 g for 5 min and the supernatants were transferred into their corresponding flasks. After a third extraction, which was identical to the second one, solvents were evaporated using a rotary evaporator (40 °C). The remaining residue was taken up in 12 mL of water before further clean-up of the extracts. For that, Discovery® DSC-18 cartridges (500 mg/6 mL, Sigma-Aldrich, Bellefonte, PA, USA) were preconditioned with 6 mL MeOH and 6 mL of water, followed by loading the sample onto the column. After two washing steps, one with 6 mL H_2_O and one with 6 mL ACN/H_2_O (1.5/8.5, v/v), analytes were eluted with 6 mL MeOH and 9 mL MeOH/2% NH_4_OH, successively. All solvents were evaporated using a rotary evaporator (40 °C). Samples were taken up in 1 mL ACN/H_2_O (3/7, v/v), membrane-filtered (PVDF, 0.2 µm), and stored at − 18 °C until LC–MS/MS measurements.

### LC–MS/MS analysis

Liquid chromatography was conducted on a Shimadzu Nexera X2 UHPLC system (Shimadzu, Kyoto, Japan) as previously published (Scheibenzuber et al. 2021). Chromatographic separation was performed on a Hyperclone BDS C18 column (150 × 3.2 mm, 3 µm, 130 Å, Phenomenex, Aschaffenburg, Germany) for all toxins except TeA, which was analyzed separately on a Gemini-NX C18 column (150 × 4.6 mm, 3 µm, 110 Å, Phenomenex, Aschaffenburg, Germany). The binary gradient for the multi-mycotoxin method was set as follows: 0–2 min 10% B, 2–2.5 min 10–18% B, 2.5–10.5 min 18% B, 10.5–14 min 18–40% B, 14–20 min 40% B, 20–23 min 40–100% B, 23–25 min 100% B, 25–27 min 100–10% B, and 27–32 min 10% B. The flow rate was set to 0.3 mL/min, solvents were water (A) and acetonitrile (B), the injection volume was 10 µL, and the column oven was tempered to 40 °C. For the analysis of TeA, solvent A was a 5 mM ammonium formate solution (adjusted to pH 9 with ammonia solution), and solvent B was methanol as published previously (Asam et al. 2013). The flow rate was 0.5 mL/min, the injection volume 10 µL, and the oven temperature was set to 40 °C. Here, the binary gradient was as follows: 0–3 min 5% B, 5–8 min 5–100% B, 8–10 min 100% B, 10–13 min 100–5% B, and 13–24 min 5% B. Due to fourfold solvent selection for each pump and a column oven for up to six columns, both methods could be run in sequence by using the automated column switching function.

The LC system was interfaced with a Shimadzu 8050 triple quadrupole mass spectrometer (Shimadzu Corporation, Kyoto, Japan). The ion source parameters are listed in the following: heat block temperature 400 °C, interface temperature 300 °C, desolvation temperature 250 °C, interface voltage 4 kV, drying gas flow 10 L/min, heating gas flow 10 L/min, nebulizing gas flow 3 L/min, and collision-induced dissociation gas pressure 270 kPa. All measurements were operated in the negative electrospray ionization (ESI) mode and the multiple reaction monitoring (MRM) mode was used. To optimize MS parameters for each analyte, standard solutions of every toxin (0.01 µg/mL to 1 µg/mL) were directly injected into the source. From the six optimized mass transition, the two most dominant ones were chosen for quantification and for identification. Mass transitions for each toxin as well as their retention times, final collision energies, and optimized voltages are listed in Table 1. For data acquisition and data analysis, the LabSolutions Software (Shimadzu, Kyoto, Japan) was used.

**Table 1:** LC–MS/MS parameters of the analyzed *Alternaria* mycotoxins

Analyte	Precursor ion *m/z*	Product ion *m/z*	Q1 pre-bias [V]	CE [V]	Q3 pre-bias [V]	Rt [min]
AOH	256.9	213.15	18	23	20	18.44 ± 0.04
		212.10	48	29	38	
[^2^H_4_]-AOH	260.9	217.15	18	23	20	18.40 ± 0.03
		216.10	48	29	38	
AME	271.1	256.10	20	23	24	24.13 ± 0.01
		255.10	20	31	24	
[^2^H_4_]-AME	275.1	260.10	20	23	24	24.11 ± 0.02
		259.10	20	31	24	
ALT	291.1	203.20	30	35	18	16.74 ± 0.02
		248.15	24	27	14	
ALTP	349.1	261.20	26	30	26	18.52 ± 0.02
		303.20	26	22	18	
ATX I	351.1	315.15	26	18	18	18.12 ± 0.03
		297.15	26	28	18	
ATX II	349.1	313.20	16	18	20	22.56 ± 0.02
		330.15	26	26	18	
STTX III	347.1	329.15	12	20	20	23.38 ± 0.03
		301.10	16	35	30	
TEN	413.4	141.05	14	23	12	19.32 ± 0.02
		214.25	14	26	20	
TeA	196.3	139.00	14	22	11	8.16 ± 0.03
		112.05	22	26	20	
[^13^C_6_^15^N]-TeA	203.3	142.00	14	22	11	18.14 ± 0.01
		113.05	22	26	20	
AOH-3-S	337.0	257.15	24	26	26	5.62 ± 0.21
		213.15	24	39	20	
AOH-3-G	419.1	256.15	30	33	26	14.82 ± 0.05
		228.20	30	45	12	
AOH-9-G	419.1	283.30	12	30	32	14.22 ± 0.04
		256.15	18	35	28	
AME-3-S	351.2	271.20	12	23	26	8.98 ± 0.04
		256.15	12	35	24	
AME-3-G	433.0	270.20	16	33	18	17.38 ± 0.02
		227.10	12	54	20	

### Calibration and quantitation

For toxins that were quantified by SIDA, response curves were measured by mixing constant amounts of the stable isotope–labelled standards (S) [^2^H_4_]-AOH, [^2^H_4_]-AME and [^13^C_6_,^15^ N]-TeA with different amounts of their non-labelled forms (A) to obtain molar ratios in the range from 0.001 to 100 (1:100, 1:50, 1:20, 1:10, 1:5, 1:2, 1:1, 2:1, 5:1, 10:1, 50:1, and 100:1). After measuring those standard mixtures with the LC–MS/MS method, peak area ratios [A(A)/A(S)] were calculated and plotted against the corresponding molar ratios [n(A)/n(S)] to obtain a response function after linear regression of the data.

For all other toxins, matrix-matched calibration curves were measured, using a mycotoxin-free barley sample from this study. Here, 8–10 matrix calibration points were prepared for each toxin, ranging from 2 to 10 µg/kg (ATX I), 2.2 to 22 µg/kg (ALTP), 0.2 to 13 µg/kg (TEN), 3.6 to 17.5 µg/kg (ATX II), 2.2 to 20 µg/kg (AOH-3-S), 1.5 to 15 µg/kg (AME-3-S), 3 to 20 µg/kg (AOH-3-G), 4 to 20 µg/kg (AOH-9-G), and 4.9 to 15 µg/kg (AME-3-G). After measuring all points with LC–MS/MS, peak areas [A(A)] were plotted against the concentration of the analytes [c(A)], and calibration curves were calculated by linear regression. Mandel’s fitting test was applied to all calibration curves to check for linearity (Mandel and Mansfield 1964).

For quantification of the barley and malt samples, toxin concentrations were either calculated by the respective response curves of AOH, AME, or TeA, or by matrix-matched calibration. To compensate for day to day variations and intensity variabilities of the LC–MS/MS system, two matrix calibration points were prepared with each sample batch, which were then used to check for validity of the priorly determined calibration curves. In addition, all samples were prepared in pairs, meaning that both barley and its respective malt were analyzed on the same day to ensure that both samples were measured with the same instrumental conditions.

### Method validation

#### LODs and LOQs

Limits of detection (LODs) and limits of quantifications (LOQs) were determined as described by Vogelgesang and Hädrich (1998). Therefore, an *Alternaria* toxin–free barley sample was used as blank matrix, which was spiked with the unlabelled analytes in four different concentration levels, each in triplicate (see Table 1 in the supplementary material for detailed information). Isotope-labelled standards were added and samples were further prepared for LC–MS/MS measurements following the developed sample workup procedure.

#### Precision

The blank matrix was spiked in triplicate with all analytes (see Table 1 in the supplementary material for detailed information), and was then subjected to sample preparation for intra-day (*n* = 3) and inter-day (*n* = 9, triplicate measurement every week within 3 weeks) precision measurements.

#### Recovery

Three different concentration levels of each toxin were spiked in triplicate into the blank matrix (see Table 1 in the supplementary material for detailed information). After sample preparation and LC–MS/MS measurement, recovery was calculated as ratio of the found value divided through the spiked value times 100.

#### Sample homogeneity

To check for homogeneity of the sampling procedure, the following experiment was performed: three CBS were chosen and from two respective LBS thereof the MABS were prepared, extracted in duplicate and analyzed with double injections. The variation of the complete sample set was calculated for each toxin. The same scheme was followed for MAMS from the same CBS.

## Results and discussion

### Sample preparation

Sample preparation was based on a method described by Gotthardt et al. (2019b). However, some adaptions had to be made for the integration of the modified *Alternaria* toxins into the method. First, extraction solvents containing methanol were avoided, as more matrix components were extracted with methanol-based than with acetonitrile-based extraction solvents. Also, the addition of 0.02% formic acid reduced the capability to extract modified toxins, while at the same time showing the highest recoveries for AOH, AME, ATX I, and ALTP. Therefore, the first extraction step was conducted with acetonitrile/water (84/16, v/v) without acid to mainly extract the modified mycotoxins, while a second and third extraction was done with acetonitrile/water (84/16, v/v) and 0.02% formic acid for better recoveries of AOH, AME, ATX I, and ALTP. The usage of the azeotropic mixture acetonitrile/water (84/16, v/v) facilitated the evaporation in the following step. To prevent signal suppression in the ESI source and contamination of the LC–MS/MS by matrix components, a purification step using solid-phase extraction (SPE) was necessary. Using C18 material was effective for matrix removal in accordance to literature (Asam et al. 2011b, 2012; Gotthardt et al. 2019b; Scheibenzuber et al. 2021); however, some alterations had to be made for barley and malt matrices. Here, especially the second washing step with ACN/H_2_0 (1.5/8.5, v/v) reduced the matrix burden due to the increased polarity, while at the same time not being polar enough to elute the modified toxins from the column.

### Calibration and quantitation

Linearity of the calculated response functions of AOH, AME, and TeA towards their corresponding isotope-labelled standards was checked with Mandel’s fitting test (Mandel and Mansfield 1964) and was confirmed for molar ratios between 0.01 and 100 for all three toxins.

Matrix-matched calibration curves were obtained for all other analytes by spiking a blank matrix with at least eight different concentrations. Here, the lowest spiking level was identical to the LOQ, while the highest concentration level was at least ten times higher than the LOQ. Again, linearity was checked with Mandel’s fitting test, followed by reducing the chosen range when necessary, resulting in the following linear ranges: 0.4–13 µg/kg for TEN, 2.1–10 µg/kg for ATX I, 3.6–17.5 µg/kg for ATX II, 2.1–22 µg/kg for ALTP, 8.8–100 µg/kg for ALT, 2.2–20 µg/kg for AOH-3-S, 2.2–15 µg/kg for AME-3-S, 2.9–20 µg/kg for AOH-3-G, 3.9–20 µg/kg for AOH-9-G, and 4.9–15 µg/kg AME-3-G.

### Method validation

Method validation was performed as suggested by Vogelgesang and Hädrich (1998). All results are summarized in Table 2. An analyte-free barley sample was spiked in triplicate with four different concentration levels to determine the limits of detections (LODs) and limits of quantification (LOQs). In this matrix, LOD values were between 0.05 µg/kg (AME) and 2.45 µg/kg (ALT), while LOQ values ranged from 0.16 µg/kg (AME) to 8.75 µg/kg (ALT). Measuring ALT in the ESI positive mode would have improved the sensitivity and most methods described in literature (Nguyen et al. 2018; Wang et al. 2016) do so. However, as all other toxins were most sensitive in the negative ESI mode and we did not want to measure ALT separately, we had to compromise and accept the higher LODs and LOQs for this analyte. Anyway, LODs and LOQs of other methods measuring ALT in ESI negative mode are similar to the values in our study (Puntscher et al. 2018).

**Table 2:** Limits of detection (LODs), limits of quantitation (LOQs), relative standard deviation (RSD) values and recoveries for 13 *Alternaria* toxins in barley. Recovery values of each spiking level were determined as mean value of three replicates and triple injections

Analyte	LOD	LOQ	Precision (RSD) [%]			Recovery [%]		
	[µg/kg]	[µg/kg]	Inter-injection (*n* = 5)	Intra-day (*n* = 3)	Inter-day (*n* = 9)	Level 1	Level 2	Level 3
AOH	0.5	1.6	2	2	3	96 ± 2	97 ± 4	95 ± 1
AME	0.05	0.16	2	2	3	107 ± 4	100 ± 1	98 ± 3
TeA	0.6	2.3	2	4	5	96 ± 3	99 ± 5	96 ± 4
TEN	0.1	0.4	4	5	9	104 ± 5	106 ± 3	112 ± 3
ATX I	0.7	2.1	4	7	9	93 ± 7	92 ± 12	93 ± 8
ATX II	1.0	3.6	5	5	10	90 ± 3	89 ± 5	95 ± 2
ALTP	0.6	2.1	5	7	5	90 ± 4	92 ± 7	94 ± 6
ALT	2.5	8.8	4	9	10	84 ± 2	92 ± 7	94 ± 7
AOH3G	0.8	3.0	4	4	4	93 ± 1	91 ± 3	87 ± 8
AOH9G	1.0	3.9	5	5	6	84 ± 2	85 ± 4	91 ± 4
AME3G	1.6	4.9	3	5	9	95 ± 1	97 ± 4	98 ± 2
AOH3S	0.5	2.2	4	6	6	87 ± 2	106 ± 7	103 ± 7
AME3S	0.6	2.2	5	9	10	103 ± 2	98 ± 2	93 ± 4

To determine the recovery of every analyte, a blank matrix was spiked in triplicate with three different concentrations that ideally resembled the expected concentrations in the samples. As in real samples low or no mycotoxin contaminations were expected, the first level was the concentration of the LOQ, while the other two levels were chosen close to the first level. Only for TeA spiking concentrations up to 500 µg/kg were used as higher levels were expected for this toxin. Recoveries for all analytes laid within the acceptable range of 70 to 120% (Table 2), mean value of the lowest recovery was 84% for ALT and AOH-9-G, and the mean value of the highest recovery was 112% for TEN.

Inter-injection, intra-day, and inter-day precisions were determined as the relative standard deviation (RSD) of every analyte after a certain number of repeated measurements. All obtained precisions are listed in Table 2. For inter-injection precision measurements, one spiked sample was measured five times in a row with LC–MS/MS, resulting in precisions ranging from 2 to 5%, which demonstrates the stability of the used system. To determine the intra-day precision, one sample was prepared in triplicate, while for inter-day precisions one sample was prepared in triplicate every week within 3 weeks. RSD values for both were below 10%, which shows the good precision of the used method.

### Sample homogeneity

The analysis of two respective individual LBS and MBS from the same CBS showed reasonable variations in the toxin contents, demonstrating the suitability of the used sampling method for this study. For AOH, ATX I and ALTP variations in these real samples were lower than 10% and are therefore comparable to the obtained values for inter-day precisions from the method validation with spiked samples. AME and TEN, both of which were often present in concentrations below 2 µg/kg, showed maximum variations of less than 30%, and TeA concentrations varied up to 20% for concentrations close to the LOQ, and less than 12% for concentrations over 10 µg/kg (see Table 2 in the supplementary information for more details). We concluded that sample heterogeneity could be handled satisfactorily within the general uncertainty of analytical methods in trace analysis, therefore.

### Screening of 50 barley and malt samples

Fifty malting barley samples were analyzed before and after malting to get a first insight into *Alternaria* mycotoxin occurrence and formation during malting. An overview of the obtained results is shown in Table 3 and the complete dataset can be found in Table 5 in the supplementary information.

**Table 3:** Summary of the results of the analysis of 50 barley and malt samples. Sample concentrations are given as the mean value of two replicates and double injections. The toxins ALT, ATX II, AME-3-S, AOH-3-G, AOH-9-G, and AME-3-G were not detected in any sample

	Samples > LOD (*n* = 50)		Samples > LOQ (*n* = 50)		Lowest Concentration (µg/kg)		Highest concentration (µg/kg)	
	Barley	Malt	Barley	Malt	Barley	Malt	Barley	Malt
AOH	13	23	11	15	1.91 ± 0.07	1.87 ± 0.15	20.60 ± 1.2	15.6 ± 1.3
AME	9	13	9	13	1.16 ± 0.04	1.06 ± 0.06	6.62 ± 0.66	6.53 ± 0.4
TeA	42	48	29	45	2.52 ± 0.14	3.42 ± 0.33	165 ± 9	247.1 ± 16.2
ALTP	5	30	3	15	4.24 ± 0.38	2.95 ± 0.12	6.73 ± 0.44	15.56 ± 2.54
ATX I	7	24	2	4	3.12 ± 0.49	2.40 ± 0.19	3.12 ± 0.23	4.19 ± 0.44
TEN	29	29	15	13	0.54 ± 0.06	0.46 ± 0.04	3.09 ± 0.52	2.35 ± 0.06
AOH3S	1	3	1	3	6.23 ± 0.49	3.18 ± 0.2	6.23 ± 0.49	15.66 ± 0.91

Out of all analyzed samples, only five barley samples and one malt sample were completely free of *Alternaria* toxins; the other samples were contaminated with at least one toxin.

For barley, 42 samples were contaminated with TeA with concentrations up to 165 ± 9 µg/kg. TEN was found in 29 and AOH in 13 barley samples. The toxins AME, ALTP, and ATX I were detected in less than ten samples and the modified toxin AOH-3-S could only be found in one sample. Generally, concentrations were rather low or even below the LOQ, and the samples with the highest concentration of each toxin do not seem critical in terms of exposure. Interestingly, samples from the 2017 harvest were noticeably more often contaminated than the samples from the other three harvest years, with every sample containing at least three *Alternaria* mycotoxins. A more detailed list with data for every harvest year can be found in the supplementary information (Tables 3 and 4).

In general, toxins that were detected in the barley samples were also found in their respective malts with only four exceptions in sample pairs no. 20, 22, 26, and 43 regarding the toxins ATX I, TEN, TeA, and TEN, respectively. In the latter case, concentrations of the respective toxins in all sample pairs except sample no. 20 were below the LOQ values in barley and the absence in malt could therefore be attributed to measurement uncertainty at low concentrations. However, in sample no. 20, 3.12 µg/kg ATX I could be found in barley, but was not detected in the respective malt. Chemical or enzymatic modification of this toxin during the malting process may be an explanation for this finding.

In total, more malt than barley samples showed contaminations with *Alternaria* toxins, which indicates a mycotoxin formation during the malting process. Especially the number of samples containing ALTP and ATX I was noticeably higher with 30 and 24 positive (> LOD) malt samples, respectively, compared to 5 and 7 positive (> LOD) barley samples, respectively. However, half of the malt samples containing ALTP and almost all malt samples containing ATX I showed concentrations below the LOQ. Thus, for these two toxins, a clear evidence of formation during malting is hampered by measurement uncertainty, unfortunately. However, the increase of samples exceeding the LOD for ALTP and ATX I after malting seems to be more than a coincidence. As well as in barley, tenuazonic acid was the most dominant toxin in the malt samples and was detected in 48 out of 50 samples. However, an increase in TeA concentration was observed in almost every sample this time, reaching up to 247 µg/kg TeA as the maximal amount. For AOH, the number of contaminated samples increased from 13 to 23 after malting, and AME was found in 13 malt samples instead of 9 barley samples, but overall concentrations of AOH and AME stayed relatively stable or were only slightly higher in malt, which indicates only a low formation of both toxins during malting. For TEN, the number of contaminated barley and malt samples was rather stable, and concentrations did not show any major differences between the raw material and the malt.

**Fig. 2:**
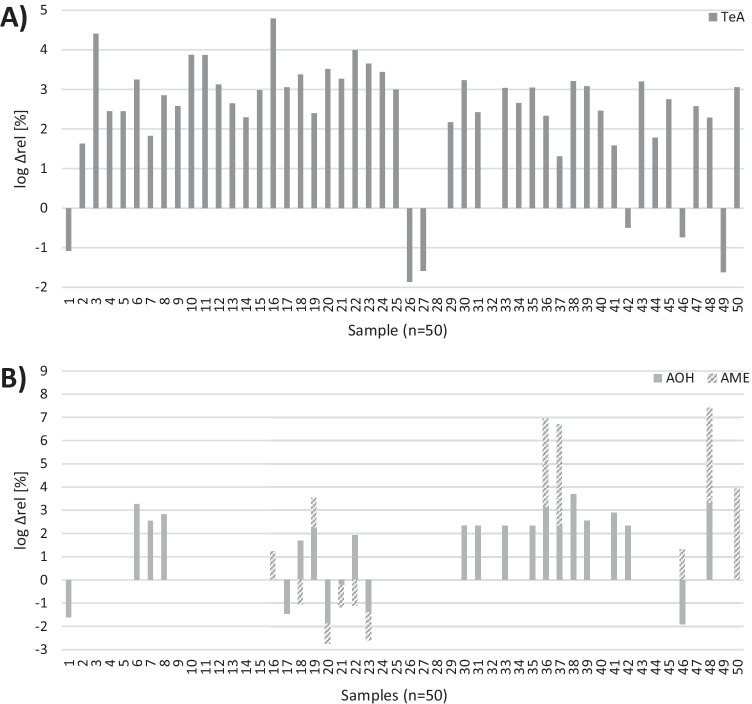
Graphic visualization of the mycotoxin behavior of TeA **A**) and the sum of AOH and AME **B**) in barley and malt, calculated as the relative increase/decline of mycotoxins in malt, using mean values of each sample: ${\Delta }_{\mathrm{rel}}=\frac{{\overline{c} }_{\mathrm{malt}}-{\overline{c} }_{\mathrm{barley}}}{{\overline{c} }_{\mathrm{barley}}}\times 100$. A logarithmic scale was used for better display of data

Figure 2 shows the relative increase or decline of the toxins TeA, AOH, and AME of every malt sample. We chose a relative presentation of the data because of the different toxin contents of the barley samples and a logarithmic scale because of the large concentration differences. For TeA, an increase was observed in 44 samples, whereas six samples showed a reduced TeA concentration in malt. A hypothesis for the decrease of these six samples could be that more TeA was removed with the steeping water due to its high polarity than was formed by the *Alternaria* fungus. However, the steeping water was not analyzed in this study and more detailed studies of the malting process are required to confirm this theory, therefore. For AOH and AME, an increase in concentration was observed for 18 and 7 samples, respectively, and a decrease for 6 and 8 samples, respectively. Interestingly, the AOH concentration in sample no. 46 decreased from 11.89 µg/kg in barley to 6.94 µg/kg in malt while at the same time the modified toxin AOH-3-S increased from 6.23 µg/kg to 15.66 µg/kg. This indicates a transformation process during malting and a potential reason for the toxin decrease. However, two barley samples did neither contain AOH nor AOH-3-S, but both toxins were found in the respective malt samples, which shows that a simultaneous formation of both toxins during malting is also possible. In the malt samples that showed a decreased AME content, no AME-3-S or AME-3-G could be detected, but as the concentrations of AME were rather low, it could be possible that formed modified toxins were below the LOD and therefore not detected. Also, the formation of modified *Alternaria* toxins other than glucosides and sulfates cannot be excluded at this point.

Our results confirmed the hypothesis that *Alternaria* mycotoxin formation may take place during malting as the number of contaminated samples increased for almost every toxin. We did not perform significance tests at sample level due to limited data (duplicate analyses). However, to validate our observations, a one-tailed paired *t*-test was performed for all samples and all mycotoxins to see if mycotoxin concentrations are significantly higher in malt than in barley in general. For calculation, the middle-bound scenario for left-censored data was applied; i.e., concentrations of ½ LOD were used for all non-detected analytes (< LOD), and concentrations of ½ LOQ were used for all values between LOD and LOQ. The test revealed significantly higher concentrations (*α* = 0.05) for the mycotoxins TeA, AOH, AME, ATX I, and ALTP, which means that those toxins are most likely to be formed during malting. No significant difference (*α* = 0.05) could be found for the toxins TEN and AOH-3-S, which also confirmed our previous observations. Those results are also mostly in accordance with a study from Prusova et al. (2021), where AOH, AME, TEN, and TeA were included in a multi-mycotoxin mycotoxin method and analyzed during malting and brewing.

Our study revealed that concentrations of AOH, AME, TeA, ALTP, and ATX I were higher in malt than in barley, which indicates mycotoxin formation during malting. Especially the formation of ALTP and ATX I, which were only rarely found in barley, seems to be promoted by the conditions during malting. However, concentrations of all detected toxins were generally low and often below the LOQ in both barley and malt. Anyway, more data is needed for further risk assessments as fungal growth and mycotoxin concentrations in barley could be elevated in harvest years with unfortunate climate that leads to increased *Alternaria* infection in the field.

## Supplementary Information

Below is the link to the electronic supplementary material.

Supplementary file1 (DOCX 38 KB)

## Data Availability

The datasets generated during and analyzed during the current study are available from the corresponding author on reasonable request.
