# Supplementary material for: Development of analytical methods to study the effect of malting on levels of free and modified forms of Alternaria mycotoxins in barley
Source: Mycotoxin Res. 2022 Apr 8;38(2):137–46. doi: 10.1007/s12550-022-00455-1 (PMC9038834; doi:10.1007/s12550-022-00455-1)
Supplement: Supplementary file 1 — Supplementary file1 (DOCX 38 KB) [file 12550_2022_455_MOESM1_ESM.docx]

**Supplementary Information**

**Development of analytical methods to study the effect of malting on levels of free and modified forms of *Alternaria* mycotoxins in barley**

Sophie Scheibenzuber^1^, Fabian Dick^1^, Marina Bretträger^2^, Martina Gastl^2^, Stefan Asam^1^ and Michael Rychlik^1,^ *

^1^Chair of Analytical Food Chemistry, Department of Life Science Engineering, School of Life Sciences, Technical University of Munich, Freising, Germany

^2^Chair of Brewing and Beverage Technology, Department of Life Science Engineering, School of Life Science, Technical University of Munich, Freising, Germany

*Corresponding author:

Sophie Scheibenzuber

Chair of Analytical Food Chemistry

Maximus-von-Imhof Forum 2

85354 Freising

Germany

[sophie.scheibenzuber@tum.de](mailto:sophie.scheibenzuber@tum.de)

**1. Spiking levels used for method validation**

Table 1. Spiking levels used for the determination of LOD/LOQ, Precision and recovery of each *Alternaria* toxin.

|  | LOD and LOQ in µg/kg | | | |  | Precision in µg/kg |  | Recovery in µg/kg | | |
| --- | --- | --- | --- | --- | --- | --- | --- | --- | --- | --- |
|  | Level 1 | Level 2 | Level 3 | Level 4 |  | Level 1 |  | Level 1 | Level 2 | Level 3 |
| AOH | 0.5 | 2.5 | 4 | 5.5 |  | 4.0 |  | 2.5 | 4.0 | 5.5 |
| AME | 0.05 | 0.30 | 0.75 | 1.5 |  | 0.3 |  | 0.3 | 0.75 | 1.5 |
| TeA | 1.2 | 4.5 | 7.0 | 10.0 |  | 100 |  | 10 | 100 | 500 |
| TEN | 0.1 | 0.7 | 1.0 | 2.0 |  | 1.5 |  | 0.7 | 1.0 | 2.0 |
| ATX I | 0.3 | 1.2 | 2.0 | 3.5 |  | 8.0 |  | 2.1 | 3.5 | 8 |
| ATX II | 0.2 | 1.0 | 2.5 | 5 |  | 5.0 |  | 3.6 | 5.0 | 7.0 |
| ALTP | 0.25 | 1.0 | 2.0 | 5.0 |  | 3.5 |  | 2.1 | 3.5 | 5 |
| ALT | 3.0 | 13.0 | 16.0 | 20.0 |  | 16 |  | 13 | 26 | 18 |
| AOH3S | 0.5 | 2.5 | 4.0 | 5.5 |  | 5.5 |  | 2.5 | 4.0 | 5.5 |
| AME3S | 0.5 | 2.5 | 4.0 | 5.5 |  | 8.0 |  | 4.0 | 5.5 | 8.0 |
| AOH3G | 1.0 | 5.0 | 7.5 | 9.0 |  | 4.0 |  | 5.0 | 7.5 | 9.0 |
| AOH9G | 0.5 | 3.0 | 5.0 | 7.5 |  | 9.0 |  | 7.5 | 9.0 | 12 |
| AME3G | 0.5 | 5.0 | 6.0 | 7.5 |  | 6.0 |  | 5.0 | 6.0 | 7.5 |

**2. Sample Homogeneity**

Table 2. Results of the homogeneity analysis of the used sampling method. Var % = coefficient of variation in % between two separately obtained MABS and MAMS. Samples were prepared in duplicates with double injections.

| sample | | AOH | |  | AME | |  | TeA | |  | TEN | |  | ALTP | |  |
| --- | --- | --- | --- | --- | --- | --- | --- | --- | --- | --- | --- | --- | --- | --- | --- | --- |
|  |  | µg/kg | Var % |  | µg/kg | Var % |  | µg/kg | Var % |  | µg/kg | Var % |  | µg/kg | Var % | |
| B46 | MABS 1 | 11.9 ± 0.9 | 3 |  | 5.39 ± 0.17 | 4 |  | 165 ± 9 | 10 |  | 0.77 ± 0.13 | 20 |  | 4.53 ± 0.47 | 8 | |
|  | MABS 2 | 11.4 ± 0.4 |  |  | 5.59 ± 0.42 |  |  | 149 ± 11 |  |  | 0.62 ± 0.27 |  |  | 4.89 ± 0.58 |  | |
|  |  |  |  |  |  |  |  |  |  |  |  |  |  |  |  | |
| B48 | MABS 1 | n.d. | n.d. |  | n.d. | n.d. |  | 15.3 ± 0.9 | 12 |  | 1.65 ± 0.24 | 2 |  | n.d. | n.d. | |
|  | MABS 2 | n.d. |  |  | n.d. |  |  | 13.4 ± 1.6 |  |  | 1.68 ± 0.07 |  |  | n.d. |  | |
|  |  |  |  |  |  |  |  |  |  |  |  |  |  |  |  | |
| B50 | MABS 1 | n.d. | n.d. |  | n.d. | n.d. |  | 3.33 ± 0.14 | 22 |  | 0.57 ± 0.09 | 20 |  | n.d. | n.d. | |
|  | MABS 2 | n.d. |  |  | n.d. |  |  | 2.59 ± 0.16 |  |  | 0.46 ± 0.06 |  |  | n.d. |  | |
|  |  |  |  |  |  |  |  |  |  |  |  |  |  |  |  | |
| M46 | MAMS 1 | 6.94 ± 0.33 | 0 |  | 6.53 ± 0.40 | 3 |  | 156 ± 11 | 10 |  | 1.43 ± 0.27 | 13 |  | <LOQ | n.d. | |
|  | MAMS 2 | 6.93 ± 0.12 |  |  | 6.35 ± 0.19 |  |  | 141 ± 13 |  |  | 1.61 ± 0.22 |  |  | <LOQ |  | |
|  |  |  |  |  |  |  |  |  |  |  |  |  |  |  |  | |
| M48 | MAMS 1 | 2.44 ± 0.28 | 2 |  | 3.18 ± 0.54 | 3 |  | 45.2 ± 3.5 | 12 |  | 0.83 ± 0.12 | 13 |  | 3.35 ± 0.38 | 2 | |
|  | MAMS 2 | 2.50 ± 0.46 |  |  | 3.09 ± 0.52 |  |  | 39.70 ± 5.1 |  |  | 0.72 ± 0.10 |  |  | 3.40 ± 0.27 |  | |
|  |  |  |  |  |  |  |  |  |  |  |  |  |  |  |  | |
| M50 | MAMS 1 | n.d. | n.d. |  | 2.15 ± 0.16 | 28 |  | 41.3 ± 2.0 | 2 |  | 0.59 ± 0.11 | 28 |  | <LOQ | n.d. | |
|  | MAMS 2 | n.d. |  |  | 1.56 ± 0.10 |  |  | 41.9 ± 3.9 |  |  | 0.42 ± 0.04 |  |  | <LOQ |  | |

**3. Mycotoxin concentrations in barley from different harvest years**

Table 3. Number of contaminated samples, as well as minimal and maximal concentrations of seven *Alternaria* mycotoxins found in 50 barley samples from five consecutive harvest years.

|  | AOH | AME | TeA | ALTP | ATX I | TEN | AOH3S |
| --- | --- | --- | --- | --- | --- | --- | --- |
| **samples > LOD** |  |  |  |  |  |  |  |
| **over 5 years (n=50)** | **13** | **9** | **42** | **5** | **7** | **29** | **1** |
| *in 2016 (n=15)* | *3* | *0* | *10* | *1* | *0* | *3* | *0* |
| *in 2017 (n=8)* | *8* | *8* | *7* | *0* | *7* | *8* | *0* |
| *in 2018 (n=6)* | *0* | *0* | *6* | *0* | *0* | *2* | *0* |
| *in 2019 (n=15)* | *1* | *0* | *13* | *2* | *0* | *11* | *0* |
| *in 2020 (n=6)* | *1* | *1* | *6* | *2* | *0* | *5* | *1* |
| **samples < LOQ** |  |  |  |  |  |  |  |
| **over 5 years (n=50)** | **2** | **0** | **13** | **2** | **5** | **14** | **0** |
| *in 2016 (n=15)* | *2* | *0* | *3* | *0* | *0* | *1* | *0* |
| *in 2017 (n=8)* | *0* | *0* | *4* | *0* | *5* | *7* | *0* |
| *in 2018 (n=6)* | *0* | *0* | *3* | *0* | *0* | *1* | *0* |
| *in 2019 (n=15)* | *0* | *0* | *2* | *2* | *0* | *5* | *0* |
| *in 2020 (n=6)* | *0* | *0* | *1* | *0* | *0* | *0* | *0* |
| **Lowest Concentration (µg/kg)** |  |  |  |  |  |  |  |
| **over 5 years (n=50)** | **1.91 ± 0.07** | **1.16 ± 0.04** | **2.52 ± 0.14** | **4.24 ± 0.38** | **3.12 ± 0.49** | **0.54 ± 0.06** | **6.23 ± 0.49** |
| *in 2016 (n=15)* | *3.20 ± 0.15* | *-* | *2.52 ± 0.14* | *4.24 ± 0.38* | *-* | *0.56 ± 0.08* | *-* |
| *in 2017 (n=8)* | *1.91 ± 0.07* | *1.16 ± 0.04* | *2.86 ± 0.02* | *-* | *3.12 ± 0.49* | *0.54 ± 0.06* | *-* |
| *in 2018 (n=6)* | *-* | *-* | *3.57 ± 0.34* | *-* | *-* | *0.55 ± 0.06* | *-* |
| *in 2019 (n=15)* | *2.69 ± 0.21* | *-* | *3.24 ± 0.25* | *-* | *-* | *0.5 ± 0.04* | *-* |
| *in 2020 (n=6)* | *6.94 ± 0,33* | *5.39 ± 0.17* | *2.93 ± 0.2* | *4.53 ± 0.47* | *-* | *0.57 ± 0.09* | *6.23 ± 0.49* |
| **Highest concentration (µg/kg)** |  |  |  |  |  |  |  |
| **over 5 years (n=50)** | **20.6 ± 1.2** | **6.62 ± 0.66** | **165 ± 9** | **6.73 ± 0.44** | **3.12 ± 0.23** | **3.09 ± 0.52** | **6.23 ± 0.49** |
| *in 2016 (n=15)* | *3.20 ± 0.15* | *-* | *40.5 ± 2.7* | *4.24 ± 0.38* | *-* | *0.60 ± 0.04* | *-* |
| *in 2017 (n=8)* | *20.6 ± 1.2* | *6.62 ± 0.66* | *3.19 ± 0.1* | *-* | *3.12 ± 0.23* | *0.54 ± 0.06* | *-* |
| *in 2018 (n=6)* | *-* | *-* | *8.82 ± 0.21* | *-* | *-* | *0.55 ± 0.06* | *-* |
| *in 2019 (n=15)* | *2.69 ± 0.21* | *-* | *51.2 ± 1.0* | *-* | *-* | *3.09 ± 0.52* | *-* |
| *in 2020 (n=6)* | *6.94 ± 0.33* | *5.39 ± 0.17* | *165 ± 9* | *6.73 ± 0.44* | *-* | *1.65 ± 0.24* | *6.23 ± 0.49* |

**4. Mycotoxin concentrations in malt from different harvest years**

Table 4. Number of contaminated samples, as well as minimal and maximal concentrations of seven *Alternaria* mycotoxins found in 50 malt samples from five consecutive harvest years.

|  | AOH | AME | TeA | ALTP | ATX I | TEN | AOH3S |
| --- | --- | --- | --- | --- | --- | --- | --- |
| **samples > LOD** |  |  |  |  |  |  |  |
| **over 5 years (n=50)** | **23** | **13** | **48** | **30** | **24** | **29** | **3** |
| *in 2016 (n=15)* | *4* | *0* | *15* | *9* | *8* | *3* | *2* |
| *in 2017 (n=8)* | *8* | *8* | *8* | *0* | *8* | *7* | *0* |
| *in 2018 (n=6)* | *0* | *0* | *5* | *3* | *0* | *3* | *0* |
| *in 2019 (n=15)* | *9* | *2* | *14* | *13* | *7* | *11* | *0* |
| *in 2020 (n=6)* | *2* | *3* | *6* | *5* | *1* | *5* | *1* |
| **Samples < LOQ** |  |  |  |  |  |  |  |
| **over 5 years (n=50)** | **8** | **0** | **3** | **15** | **20** | **16** | **0** |
| *in 2016 (n=15)* | *0* | *0* | *2* | *5* | *7* | *2* | *0* |
| *in 2017 (n=8)* | *1* | *0* | *0* | *0* | *6* | *5* | *0* |
| *in 2018 (n=6)* | *0* | *0* | *1* | *3* | *0* | *1* | *0* |
| *in 2019 (n=15)* | *7* | *0* | *0* | *4* | *6* | *7* | *0* |
| *in 2020 (n=6)* | *0* | *0* | *0* | *3* | *1* | *1* | *0* |
| **Lowest concentration (µg/kg)** |  |  |  |  |  |  |  |
| **over 5 years (n=50)** | **1.87 ± 0.15** | **1.06 ± 0.06** | **3.42 ± 0.33** | **2.95 ± 0.12** | **2.40 ± 0.19** | **0.46 ± 0.04** | **3.18 ± 0.2** |
| *in 2016 (n=15)* | *1.87 ± 0.15* | *-* | *3.42 ± 0.33* | *4.15 ± 0.41* | *2.40 ± 0.19* | *0.49 ± 0.06* | *3.18 ± 0.2* |
| *in 2017 (n=8)* | *2.30 ± 0.20* | *1.06 ± 0.06* | *8.55 ± 0.66* | *-* | *3.02 ± 0.36* | *0.51 ± 0.06* | *-* |
| *in 2018 (n=6)* | *-* | *-* | *4.28 ± 0.4* | *-* | *-* | *0.46 ± 0.04* | *-* |
| *in 2019 (n=15)* | *2.16 ± 0.05* | *1.57 ± 0.11* | *5.1 ± 0.14* | *2.95 ± 0.12* | *3.53 ± 0.32* | *0.49 ± 0.02* | *-* |
| *in 2020 (n=6)* | *2.45 ± 0.28* | *2.15 ± 0.16* | *7.67 ± 0.36* | *3.07 ± 0.38* | *-* | *0.59 ± 0.11* | *15.7 ± 0.9* |
| **Highest concentration (µg/kg)** |  |  |  |  |  |  |  |
| **over 5 years (n=50)** | **15.6 ± 1.3** | **6.53 ± 0.4** | **247 ± 16** | **15.6 ± 2.5** | **4.19 ± 0.44** | **2.35 ± 0.06** | **15.7 ± 0.9** |
| *in 2016 (n=15)* | *6.30 ± 0.64* | *-* | *87.0 ± 2.8* | *7.28 ± 0.70* | *2.40 ± 0.19* | *0.49 ± 0.06* | *6.72 ± 0.66* |
| *in 2017 (n=8)* | *15.6 ± 1.3* | *5.56 ± 0.34* | *187 ± 11* | *-* | *4.19 ± 0.44* | *0.54 ± 0.22* | *-* |
| *in 2018 (n=6)* | *-* | *-* | *102 ± 7* | *-* | *-* | *0.48 ± 0.04* | *-* |
| *in 2019 (n=15)* | *12.8 ± 0.4* | *5.41 ± 0.37* | *247 ± 16* | *15.6 ± 2.5* | *3.53 ± 0.32* | *2.35 ± 0.06* | *-* |
| *in 2020 (n=6)* | *5.24 ± 0.15* | *6.53 ± 0.4* | *156 ± 11* | *3.35 ± 0.38* | *-* | *1.43 ± 0.27* | *15.7 ± 0.9* |

**5. Detailed list of mycotoxin concentrations**

Table 5. Detailed results of the 50 analysed barley (B) and malt (M) samples, sorted into the different harvest years. Values are given in µg/kg as the mean value of duplicates and double injections.

|  |  | AOH | AME | TeA | TEN | ALTP | ATX I | AOH-3-S |
| --- | --- | --- | --- | --- | --- | --- | --- | --- |
| 2016 | B1 | 3.20 ± 0.15 | *n.d.* | 40.5 ± 2.7 | 0.60 ± 0.04 | *n.d.* | *n.d.* | *n.d.* |
|  | M1 | 1.87 + 0.15 | *n.d.* | 35.6 ± 2.0 | <LOQ | <LOQ | <LOQ | *n.d.* |
|  | B2 | *n.d.* | *n.d.* | 8.17 ± 0.39 | *n.d.* | *n.d.* | *n.d.* | *n.d.* |
|  | M2 | *n.d.* | *n.d.* | 11.7 ± 0.1 | *n.d.* | <LOQ | *n.d.* | *n.d.* |
|  | B3 | *n.d.* | *n.d.* | *n.d.* | *n.d.* | *n.d.* | *n.d.* | *n.d.* |
|  | M3 | *n.d.* | *n.d.* | 77.2 ± 3.6 | *n.d.* | *n.d.* | <LOQ | *n.d.* |
|  | B4 | *n.d.* | *n.d.* | *n.d.* | *n.d.* | *n.d.* | *n.d.* | *n.d.* |
|  | M4 | *n.d.* | *n.d.* | <LOQ | *n.d.* | <LOQ | *n.d.* | *n.d.* |
|  | B5 | *n.d.* | *n.d.* | *n.d.* | <LOQ | *n.d.* | *n.d.* | *n.d.* |
|  | M5 | *n.d.* | *n.d.* | <LOQ | <LOQ | *n.d.* | *n.d.* | *n.d.* |
|  | B6 | *n.d.* | *n.d.* | 4.62 ± 0.28 | *n.d.* | 4.24 ± 0.38 | *n.d.* | *n.d.* |
|  | M6 | 4.95 ± 0.27 | *n.d.* | 86.3 ± 2.0 | *n.d.* | 6.07 ± 0.61 | <LOQ | 3.18 ± 0.20 |
|  | B7 | <LOQ | *n.d.* | 4.48 ± 0.17 | *n.d.* | *n.d.* | *n.d.* | *n.d.* |
|  | M7 | 3.64 ± 0.35 | *n.d.* | 7.49 ± 0.74 | *n.d.* | 4.15 ± 0.41 | *n.d.* | *n.d.* |
|  | B8 | <LOQ | *n.d.* | 3.9 ± 0.18 | 0.56 ± 0.08 | *n.d.* | *n.d.* | *n.d.* |
|  | M8 | 6.30 ± 0.64 | *n.d.* | 31.6 ± 2.3 | 0.49 ± 0.06 | 4.78 ± 0.64 | <LOQ | 6.72 ± 0.66 |
|  | B9 | *n.d.* | *n.d.* | 5.44 ± 0.53 | *n.d.* | *n.d.* | *n.d.* | *n.d.* |
|  | M9 | *n.d.* | *n.d.* | 26.2 ± 1.2 | *n.d.* | 7.28 ± 0.70 | 2.40 ± 0.19 | *n.d.* |
|  | B10 | *n.d.* | *n.d.* | *n.d.* | *n.d.* | *n.d.* | *n.d.* | *n.d.* |
|  | M10 | *n.d.* | *n.d.* | 23.0 ± 2.3 | *n.d.* | *n.d.* | <LOQ | *n.d.* |
|  | B11 | *n.d.* | *n.d.* | <LOQ | *n.d.* | *n.d.* | *n.d.* | *n.d.* |
|  | M11 | *n.d.* | *n.d.* | 87.0 ± 2.8 | *n.d.* | <LOQ | *n.d.* | *n.d.* |
|  | B12 | *n.d.* | *n.d.* | *n.d.* | *n.d.* | *n.d.* | *n.d.* | *n.d.* |
|  | M12 | *n.d.* | *n.d.* | 4.27 ± 0.31 | *n.d.* | *n.d.* | *n.d.* | *n.d.* |
|  | B13 | *n.d.* | *n.d.* | <LOQ | *n.d.* | *n.d.* | *n.d.* | *n.d.* |
|  | M13 | *n.d.* | *n.d.* | 6.26 ± 0.45 | *n.d.* | *n.d.* | <LOQ | *n.d.* |
|  | B14 | *n.d.* | *n.d.* | 2.52 ± 0.14 | *n.d.* | *n.d.* | *n.d.* | *n.d.* |
|  | M14 | *n.d.* | *n.d.* | 3.42 ± 0.33 | *n.d.* | *n.d.* | *n.d.* | *n.d.* |
|  | B15 | *n.d.* | *n.d.* | <LOQ | *n.d.* | *n.d.* | *n.d.* | *n.d.* |
|  | M15 | *n.d.* | *n.d.* | 12.3 ± 0.7 | *n.d.* | *n.d.* | <LOQ | *n.d.* |
| 2017 | B16 | 2.40 ± 0.09 | 1.19 ± 0.12 | *n.d.* | <LOQ | *n.d.* | <LOQ | *n.d.* |
|  | M16 | 2.31 ± 0.23 | 1.39 ± 0.12 | 187 ± 11 | <LOQ | *n.d.* | <LOQ | *n.d.* |
|  | B17 | 3.23 ± 0.28 | 1.16 ± 0.09 | 3.05 + 0.26 | <LOQ | *n.d.* | 3.12 ± 0.49 | *n.d.* |
|  | M17 | 2.30 ± 0.20 | 1.16 ± 0.04 | 36.9 ± 2.1 | <LOQ | *n.d.* | <LOQ | *n.d.* |
|  | B18 | 1.91 ± 0.07 | 1.2 ± 0.07 | <LOQ | <LOQ | *n.d.* | <LOQ | *n.d.* |
|  | M18 | 2.84 ± 0.28 | 1.06 ± 0.03 | 30.2 ± 1.8 | <LOQ | *n.d.* | <LOQ | *n.d.* |
|  | B19 | 2.36 ± 0.11 | 1.25 ± 0.1 | <LOQ | 0.54 ± 0.06 | *n.d.* | <LOQ | *n.d.* |
|  | M19 | 6.58 ± 0.65 | 1.49 ± 0.10 | 8.55 ± 0.60 | 0.54 ± 0.22 | *n.d.* | <LOQ | *n.d.* |
|  | B20 | 3.00 ± 0.13 | 1.15 ± 0.08 | 2.86 ± 0.02 | <LOQ | *n.d.* | 3.12 ± 0.23 | *n.d.* |
|  | M20 | <LOQ | 1.06 ± 0.06 | 99.8 ± 8.0 | <LOQ | *n.d.* | *n.d.* | *n.d.* |
|  | B21 | 3.77 ± 0.33 | 1.5 ± 0.02 | <LOQ | <LOQ | *n.d.* | <LOQ | *n.d.* |
|  | M21 | 3.71 ± 0.36 | 1.35 ± 0.14 | 25.7 ± 1.5 | 0.51 ± 0.06 | *n.d.* | 3.02 ± 0.36 | *n.d.* |
|  | B22 | 3.24 ± 0.29 | 1.65 ± 0.07 | <LOQ | <LOQ | *n.d.* | *n.d.* | *n.d.* |
|  | M22 | 5.90 ± 0.54 | 1.43 ± 0.14 | 111 ± 11 | *n.d.* | *n.d.* | <LOQ | *n.d.* |
|  | B23 | 20.6 ± 1.2 | 6.62 ± 0.66 | 3.19 + 0.1 | <LOQ | *n.d.* | <LOQ | *n.d.* |
|  | M23 | 15.6 ± 1.3 | 5.56 ± 0.34 | 134 ± 13 | <LOQ | *n.d.* | 4.19 ± 0.44 | *n.d.* |
| 2018 | B24 | *n.d.* | *n.d.* | 3.57 ± 0.34 | *n.d.* | *n.d.* | *n.d.* | *n.d.* |
|  | M24 | *n.d.* | *n.d.* | 102 ± 7 | *n.d.* | *n.d.* | *n.d.* | *n.d.* |
|  | B25 | *n.d.* | *n.d.* | <LOQ | *n.d.* | *n.d.* | *n.d.* | *n.d.* |
|  | M25 | *n.d.* | *n.d.* | 12.7 ± 0.5 | <LOQ | *n.d.* | *n.d.* | *n.d.* |
|  | B26 | *n.d.* | *n.d.* | <LOQ | *n.d.* | *n.d.* | *n.d.* | *n.d.* |
|  | M26 | *n.d.* | *n.d.* | *n.d.* | *n.d.* | *n.d.* | *n.d.* | *n.d.* |
|  | B27 | *n.d.* | *n.d.* | 6.99 ± 0.35 | 0.55 ± 0.06 | *n.d.* | *n.d.* | *n.d.* |
|  | M27 | *n.d.* | *n.d.* | 4.28 ± 0.4 | 0.46 ± 0.04 | <LOQ | *n.d.* | *n.d.* |
|  | B28 | *n.d.* | *n.d.* | <LOQ | *n.d.* | *n.d.* | *n.d.* | *n.d.* |
|  | M28 | *n.d.* | *n.d.* | <LOQ | *n.d.* | <LOQ | *n.d.* | *n.d.* |
|  | B29 | *n.d.* | *n.d.* | 8.82 ± 0.21 | <LOQ | *n.d.* | *n.d.* | *n.d.* |
|  | M29 | *n.d.* | *n.d.* | 21.8 ± 1.0 | 0.48 ± 0.04 | <LOQ | *n.d.* | *n.d.* |
| 2019 | B30 | *n.d.* | *n.d.* | 6.98 ± 0.24 | <LOQ | *n.d.* | *n.d.* | *n.d.* |
|  | M30 | <LOQ | *n.d.* | 126 ± 4 | 2.35 ± 0.06 | 10.9 ± 1.3 | *n.d.* | *n.d.* |
|  | B31 | *n.d.* | *n.d.* | 5.68 ± 0.24 | <LOQ | *n.d.* | *n.d.* | *n.d.* |
|  | M31 | <LOQ | *n.d.* | 20.8 ± 1.6 | <LOQ | 3.42 ± 0.35 | <LOQ | *n.d.* |
|  | B32 | *n.d.* | *n.d.* | *n.d.* | *n.d.* | *n.d.* | *n.d.* | *n.d.* |
|  | M32 | *n.d.* | *n.d.* | *n.d.* | *n.d.* | < LOQ | *n.d.* | *n.d.* |
|  | B33 | *n.d.* | *n.d.* | 14.8 ± 0.4 | 1.92 ± 0.18 | *n.d.* | *n.d.* | *n.d.* |
|  | M33 | <LOQ | *n.d.* | 177 ± 5 | 2.00 ± 0.14 | 3.68 ± 0.25 | <LOQ | *n.d.* |
|  | B34 | *n.d.* | *n.d.* | <LOQ | *n.d.* | <LOQ | *n.d.* | *n.d.* |
|  | M34 | *n.d.* | *n.d.* | 6.37 ± 0.37 | *n.d.* | 3.45 ± 0.51 | *n.d.* | *n.d.* |
|  | B35 | *n.d.* | *n.d.* | 3.33 ± 0.3 | *n.d.* | *n.d.* | *n.d.* | *n.d.* |
|  | M35 | <LOQ | *n.d.* | 40.4 ± 2.6 | *n.d.* | 3.71 ± 0.3 | <LOQ | *n.d.* |
|  | B36 | *n.d.* | *n.d.* | 7.74 ± 0.53 | 1.34 ± 0.22 | <LOQ | *n.d.* | *n.d.* |
|  | M36 | 3.69 ± 0.04 | 1.57 ± 0.11 | 24.4 ± 1.5 | 0.95 ± 0.13 | 4.22 ± 0.63 | <LOQ | *n.d.* |
|  | B37 | *n.d.* | *n.d.* | 10.3 ± 0.2 | 0.50 ± 0.04 | *n.d.* | *n.d.* | *n.d.* |
|  | M37 | <LOQ | 5.41 ± 0.37 | 12.4 ± 0.6 | <LOQ | <LOQ | *n.d.* | *n.d.* |
|  | B38 | *n.d.* | *n.d.* | < LOQ | <LOQ | *n.d.* | *n.d.* | *n.d.* |
|  | M38 | 12.8 ± 0.4 | *n.d.* | 20.0 ± 2.0 | <LOQ | 3.77 ± 0.13 | <LOQ | *n.d.* |
|  | B39 | 2.69 ± 0.21 | *n.d.* | 18.9 ± 1.6 | 2.28 ± 0.22 | *n.d.* | *n.d.* | *n.d.* |
|  | M39 | 12.2 ± 0.8 | *n.d.* | 247 ± 16 | <LOQ | 15.6 ± 2.5 | <LOQ | *n.d.* |
|  | B40 | *n.d.* | *n.d.* | 12.5 ± 0.7 | <LOQ | *n.d.* | *n.d.* | *n.d.* |
|  | M40 | *n.d.* | *n.d.* | 48.6 ± 2.0 | <LOQ | <LOQ | *n.d.* | *n.d.* |
|  | B41 | *n.d.* | *n.d.* | 51.2 ± 1.0 | 1.21 ± 0.45 | *n.d.* | *n.d.* | *n.d.* |
|  | M41 | 2.16 ± 0.05 | *n.d.* | 71.0 ± 10 | <LOQ | <LOQ | *n.d.* | *n.d.* |
|  | B42 | *n.d.* | *n.d.* | 47.5 ± 3.0 | 3.09 ± 0.52 | *n.d.* | *n.d.* | *n.d.* |
|  | M42 | <LOQ | *n.d.* | 46.0 ± 2.3 | 0.49 ± 0.02 | 2.95 ± 0.12 | *n.d.* | *n.d.* |
|  | B43 | *n.d.* | *n.d.* | *n.d.* | <LOQ | *n.d.* | *n.d.* | *n.d.* |
|  | M43 | *n.d.* | *n.d.* | 5.10 ± 0.14 | *n.d.* | *n.d.* | *n.d.* | *n.d.* |
|  | B44 | *n.d.* | *n.d.* | 3.24 ± 0.25 | *n.d.* | *n.d.* | *n.d.* | *n.d.* |
|  | M44 | *n.d.* | *n.d.* | 5.20 ± 0.15 | <LOQ | *n.d.* | *n.d.* | *n.d.* |
| 2020 | B45 | *n.d.* | *n.d.* | <LOQ | 0.61 ± 0.09 | *n.d.* | *n.d.* | *n.d.* |
|  | M45 | *n.d.* | *n.d.* | 7.67 ± 0.36 | <LOQ | <LOQ | *n.d.* | *n.d.* |
|  | B46 | 11.9 ± 0.9 | 5.39 ± 0.17 | 165 ± 9 | 0.77 ± 0.13 | 4.53 ± 0.47 | *n.d.* | 6.23 ± 0.49 |
|  | M46 | 6.94 ± 0.33 | 6.53 ± 0.4 | 156 ± 11 | 1.43 ± 0.27 | <LOQ | *n.d.* | 15.7 ± 0.9 |
|  | B47 | *n.d.* | *n.d.* | 2.93 ± 0.2 | *n.d.* | *n.d.* | *n.d.* | *n.d.* |
|  | M47 | *n.d.* | *n.d.* | 14.0 ± 0.9 | *n.d.* | *n.d.* | *n.d.* | *n.d.* |
|  | B48 | *n.d.* | *n.d.* | 15.3 ± 0.9 | 1.65 ± 0.24 | *n.d.* | *n.d.* | *n.d.* |
|  | M48 | 2.44 ± 0.28 | 3.18 ± 0.54 | 45.1 ± 3.5 | 0.83 ± 0.12 | 3.35 ± 0.38 | *n.d.* | *n.d.* |
|  | B49 | *n.d.* | *n.d.* | 33.7 ± 2.0 | 1.34 ± 0.19 | 6.73 ± 0.44 | *n.d.* | *n.d.* |
|  | M49 | *n.d.* | *n.d.* | 19.5 ± 0.7 | 0.71 ± 0.08 | 3.07 ± 0.38 | *n.d.* | *n.d.* |
|  | B50 | *n.d.* | *n.d.* | 3.33 ± 0.14 | 0.57 ± 0.09 | *n.d.* | *n.d.* | *n.d.* |
|  | M50 | *n.d.* | 2.15 ± 0.16 | 41.3 ± 2.0 | 0.59 ± 0.11 | <LOQ | <LOQ | *n.d.* |
